# Phase I/II clinical trial of brentuximab vedotin for pretreated Japanese patients with CD30‐positive cutaneous T‐cell lymphoma

**DOI:** 10.1111/1346-8138.17324

**Published:** 2024-06-14

**Authors:** Yoji Hirai, Jun Sakurai, Shiho Yoshida, Takashi Kikuchi, Toshiharu Mitsuhashi, Tomoko Miyake, Taku Fujimura, Riichiro Abe, Hiroki Fujikawa, Hikari Boki, Hiraku Suga, Sayaka Shibata, Tomomitsu Miyagaki, Takatoshi Shimauchi, Eiji Kiyohara, Yoshio Kawakami, Shin Morizane

**Affiliations:** ^1^ Department of Dermatology Graduate School of Medicine, Dentistry, and Pharmaceutical Sciences, Okayama University Okayama Japan; ^2^ Center for Innovative Clinical Medicine Okayama University Hospital Okayama Japan; ^3^ Department of Dermatology Tohoku University Graduate School of Medicine Sendai Japan; ^4^ Department of Dermatology Niigata University Niigata Japan; ^5^ Department of Dermatology Tokyo University Tokyo Japan; ^6^ Department of Dermatology Hamamatsu University School of Medicine Hamamatsu Japan; ^7^ Department of Dermatology Osaka University Osaka Japan

**Keywords:** anaplastic large‐cell lymphoma, brentuximab vedotin, cutaneous T‐cell lymphoma, mycosis fungoides, peripheral T‐cell lymphoma

## Abstract

Brentuximab vedotin (BV), a conjugate of anti‐CD30 antibody and monomethyl auristatin E, has emerged as a promising treatment option for refractory CD30+ mycosis fungoides (MF) and primary cutaneous anaplastic large‐cell lymphoma (pcALCL). BV has been shown to be safe and effective in treating Hodgkin's lymphoma and peripheral T‐cell lymphoma. This multicenter, prospective, single‐arm phase I/II study evaluated the efficacy of BV in Japanese patients with CD30+ cutaneous lymphomas, namely CD30+ cutaneous T‐cell lymphoma. Participants were divided into two groups: those with CD30+ MF or pcALCL (cohort 1, *n* = 13) and those with CD30+ lymphoproliferative disorders other than those in cohort 1 (cohort 2, *n* = 3). The studied population included the full analysis set (FAS), modified FAS (mFAS), and safety analysis set (SAF). These sets were identified in cohorts 1 and 1 + 2 and labeled FAS1 and FAS2, mFAS1 and mFAS2, and SAF1 and SAF2, respectively. Each treatment cycle lasted 3 weeks, and BV was continued for up to 16 cycles after the third cycle based on treatment response. The primary endpoint was the 4‐month objective response rate (ORR4) determined by the Independent Review Forum (IRF). ORR4 was 69.2% for FAS1 and 62.5% for FAS2 (*P* < 0.0001). Secondary endpoints of ORR, assessed using the global response score (53.8% in FAS1) and modified severity‐weighted assessment tool (62.5% in FAS1), using the IRF, provided results comparable to the primary findings. The incidence of ≥grade 3 adverse events (≥15%) in SAF1 was peripheral neuropathy in three patients (23%) and fever and eosinophilia in two patients (15%). In conclusion, BV showed favorable efficacy, tolerability, and safety profile in Japanese patients with relapsed or refractory CD30+ primary cutaneous T‐cell lymphoma. The trial was registered with University Hospital Medical Information Network Clinical Trials Registry, Japan (protocol ID: UMIN000034205).

## 1 INTRODUCTION

Cutaneous T‐cell lymphoma (CTCL) is the most common subset of cutaneous lymphomas (CLs) and includes various extranodal non‐Hodgkin's lymphomas (NHLs). This diverse group is characterized by neoplastic lymphocytic cells infiltrating the cutaneous milieu without nodal or internal involvement. According to the most recent World Health Organization–European Organization for Research and Treatment of Cancer (WHO‐EORTC) classification,^1^ mycosis fungoides (MF) is the most common type of CTCL worldwide, and its prevalence in Japan is consistent with global epidemiological trends.^2^

CTCL variants have varying prognoses. Advanced‐stage MF/Sézary syndrome (MF/SS IIB‐IVB) has a poorer prognosis than early‐stage disease (MF/SS IA–IIA).^3^, ^4^, ^5^ Primary cutaneous anaplastic large‐cell lymphoma (pcALCL), a subtype of CTCL, has a slow clinical course and favorable prognosis. However, patients with pcALCL who report leg involvement or extensive limb disease have a poorer prognosis than expected.^6^, ^7^, ^8^, ^9^ Solitary tumors in pcALCL are initially treated with surgical excision and targeted radiation therapy.^1^, ^10^ However, patients with multifocal or extracutaneous disease primarily receive single or multiagent chemotherapy regimens.

Although clinical guidelines offer treatment options for CTCL,^11^, ^12^, ^13^ the heterogeneity and complexity of the disease lead to widely varying treatment recommendations, particularly for patients in advanced stages.^14^

CD30 expression has been consistently reported in CD30+ lymphoproliferative disorders (LPDs)^15^ but differs in conditions such as MF/SS, hydroa vacciniforme‐like LPDs, peripheral T‐cell lymphoma, not otherwise specified (PTCL and NOS), and adult T‐cell leukemia/lymphoma. The consistent identification of CD30 expression suggests that it is a promising target for immunotherapeutic treatment.

Brentuximab vedotin (BV), a chimeric anti‐CD30 antibody conjugated with the anti‐tubulin agent monomethyl auristatin E, has shown effectiveness in Hodgkin's lymphoma (HL) and PTCL. BV is effective in patients with CD30+ HL and systemic ALCL (sALCL)^16^, ^17^ as well as in patients with refractory MF/SS and CD30+ LPDs.^18^, ^19^, ^20^ Consequently, in Japan, BV has become an acceptable treatment option for CD30+ HL/PTCL, including sALCL. Based on the findings from the Alcanza study,^20^ BV is authorized in the United States and Europe for the treatment of relapsed or refractory CTCL. Considering these advancements, we conducted a prospective, open‐label, multicenter phase I/II clinical trial to examine the effectiveness and safety of BV in Japanese patients with CD30+ CLs, including CTCL.

## 2 METHODS

### 2.1 Study design

This prospective, single‐arm, open‐label phase I/II multicenter study was conducted at six academic centers in Japan between April 2018 and March 2021. The first cohort included adult patients (aged ≥20 years) with CD30+ MF or pcALCL who had received at least one systemic treatment or radiotherapy. The second cohort included adult patients with CD30+ CLs other than those in cohort 1 who had undergone at least one previous systemic treatment.

This study was conducted in accordance with the International Conference on Harmonization and Good Clinical Practice. The Institutional Review Board (IRB) at each participating site (see Supporting Information Table S1 for the complete list) reviewed and approved all study‐related documents. The IRB regularly evaluated the appropriateness of the study. Okayama University Hospital (No. 290901) was registered with the University Hospital Medical Information Network, Japan (protocol ID: UMIN000034205).

**FIGURE 1:**
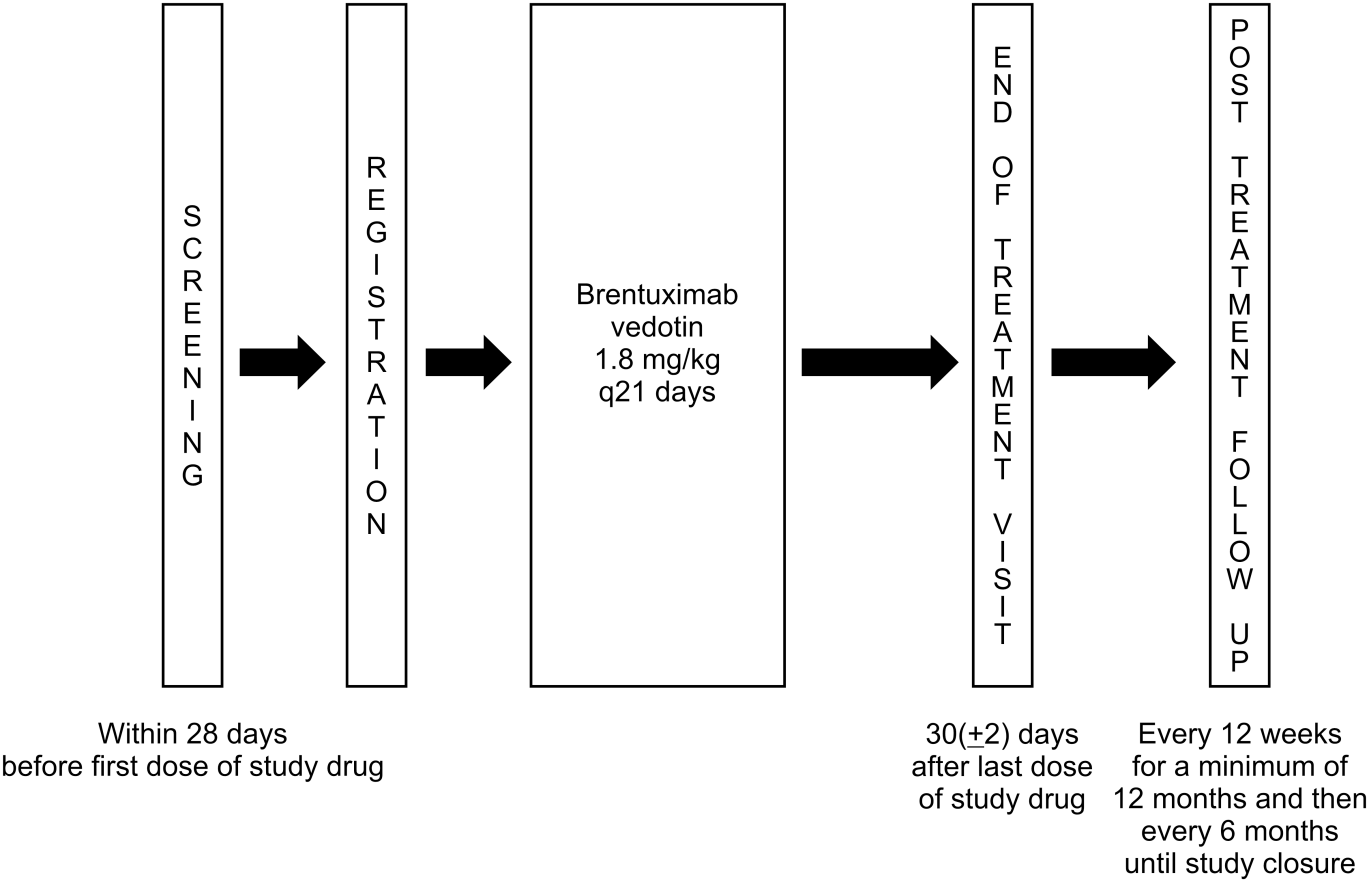
Study design.

The trial was divided into three phases: screening, treatment, and post‐treatment follow‐up (PTFU; Figure 1). Each treatment cycle was 3 weeks. Patients who completed the screening period visited the clinic every 3 weeks (21 days) for BV treatment. After three cycles, patients who showed a partial response (PR) or complete response (CR) were eligible for next phase. This repeated cycle evaluation allowed participants to receive treatment for a maximum of 16 cycles. Participants with stable disease (SD) were allowed to continue the study for up to 16 cycles at the discretion of the investigator, whereas patients with progressive disease (PD) could withdraw from the study at any time during the trial.

All participants were followed up until disease progression or the end of treatment. Participants without disease progression at end of the trial (EOT/ET) underwent PTFU and a survival study for up to 12 months after EOT/ET. Subsequently, follow‐up was performed every 6 months until disease progression, withdrawal from the study, death, or the EOT/ET. Although the study was an open‐label one, the overall efficacy evaluation conducted by the independent review forum (IRF) was blinded for the investigators and patients.

### 2.2 Study patients

The study included adults aged ≥20 years, with CD30+ lymphoproliferative disorders, including pcALCL and CD30+ MF, who had previously received prior systemic therapy and had an inadequate response, had an Eastern Cooperative Oncology Group (ECOG) performance status of ≤2, and met specific laboratory values (absolute neutrophil count, platelet count, bilirubin, aspartate transaminase, alanine transaminase, creatinine clearance, and hemoglobin). CD30+ was defined as ≥10% CD30+ malignant cells or lymphoid infiltration in one or more biopsy samples, as verified by a central review. All patients provided written informed consent before participating in the study. Patients who agreed to use contraception and had undergone adequate washout periods for previous anticancer treatments were included. Patients with a concurrent diagnosis of systemic ALCL or Sézary syndrome/B2 disease, ongoing immune therapy, recent corticosteroid treatment for CTCL, hypersensitivity to the drug or its components, current radiotherapy or other skin‐directed therapy, previous treatment with BV, and the presence of any serious medical condition determined by the investigator or clinician that may interfere with participation were excluded. A complete list of inclusion and exclusion criteria is provided in Supporting Information Table S2.

### 2.3 Treatments

Patients were administered 1.8 mg/kg BV intravenously once every 3 weeks for up to 16 3‐week cycles (Figure 1). To manage toxicity, the investigators adjusted the dose according to weight gain or loss if the weight change was >10% of the weight at screening. Only one adjustment was performed for the same participant. Treatment was continued until safety, toxicity, response, and disease progression were determined. For grade 3 treatment‐emergent adverse events (TEAEs) other than neuropathy, the drug was withheld until the patient achieved below baseline values or grade 1, and BV was resumed at the same dose and dosage after the patient recovered. Similarly, for grade 4 TEAEs, the drug was discontinued and subsequently continued at a lower dose (1.2 mg/kg) after the patient recovered. In patients with grade 2 and 3 peripheral neuropathy, the drug was discontinued and resumed at a decreased dose of 1.2 mg/kg after recovery.

### 2.4 Endpoints

According to the Alcanza study,^20^ the primary endpoint was the proportion of patients who achieved an objective global response lasting at least 4 months (ORR4). ORR4 was calculated as the percentage of participants who obtained an IRF‐determined objective response (CR or PR) lasting at least 4 months. The IRF examined the global response scores using the consensus guidelines provided by the International Society for Cutaneous Lymphoma and EORTC^4^ to calculate ORR4 and disease progression. The IRF comprised independent dermatologists who used the modified severity‐weighted assessment tool (mSWAT) to analyze and assess skin images. The secondary endpoint was the proportion of patients who achieved the response rate as defined by physicians at the implementing medical institution; skin response rate as determined by physicians at the implementing institution using mSWAT; overall survival (OS), progression‐free survival (PFS), duration of skin response, time to response, and best general effect of skin lesions as determined by physicians at the implementing institution using mSWAT; objective response lasting at least 4 weeks (ORR1) as assessed by physicians at the implementing medical institution, global response score (GRS), overall effectiveness, symptom burden, and safety based on TEAEs, and laboratory data (including hematology, blood chemistry, and urinalysis).

### 2.5 Statistical analysis

The analytical population for this study comprised the full analysis set (FAS), modified FAS (mFAS), and safety analysis set (SAF). FAS comprised participants who received at least one dose of BV. mFAS comprised participants who received at least one dose of BV, except those who tested negative for CD30. The SAF comprised participants who received at least one dose of BV. Full analysis set, mFAS, and SAF were identified in cohorts 1 and 1 + 2 and labeled FAS1 and FAS2, mFAS1 and mFAS2, and SAF1 and SAF2, respectively. We used a one‐sided exact binomial test with a 5% significance level to assess statistical significance for cohorts 1 and 1 + 2. The null hypothesis predicted no significant effect at 12% for cohort 1, whereas the working alternative hypothesis considered the observed ORR4 at 56.3%. This method yielded a power of 97.1% in detecting effects with a sample size of 12.

Due to the possibility of aggregation, the maximum number of cases in cohort 2 was limited to five. When null and alternative hypotheses common to cohorts 1 and 2 were considered, detection power for cohorts 1 + 2 (15–17 cases) was high (98.0%–99.4%). To analyze secondary endpoints, response rates were determined by estimating proportions and their associated two‐sided confidence intervals (CIs). Summary statistics, such as frequency, proportion, and two‐sided CIs, were computed to assess category levels. Mean values and two‐sided CIs (95% CIs) were determined at each evaluation time point. The relationship between the Dermatology Life Quality Index (DLQI) score, clinical response, and adverse events was investigated using exploratory analysis. The Medical Dictionary for Regulatory Activities, Japanese edition version 24.1, was used to classify TEAEs, and the Common Terminology Criteria for Adverse Events Version 5.0 (Japan Clinical Oncology Group Version) was used to determine the severity of TEAEs. Statistical analyses were performed using SAS version 9.3 or a more recent version.

## 3 RESULTS

### 3.1 Study population and treatment

**FIGURE 2:**
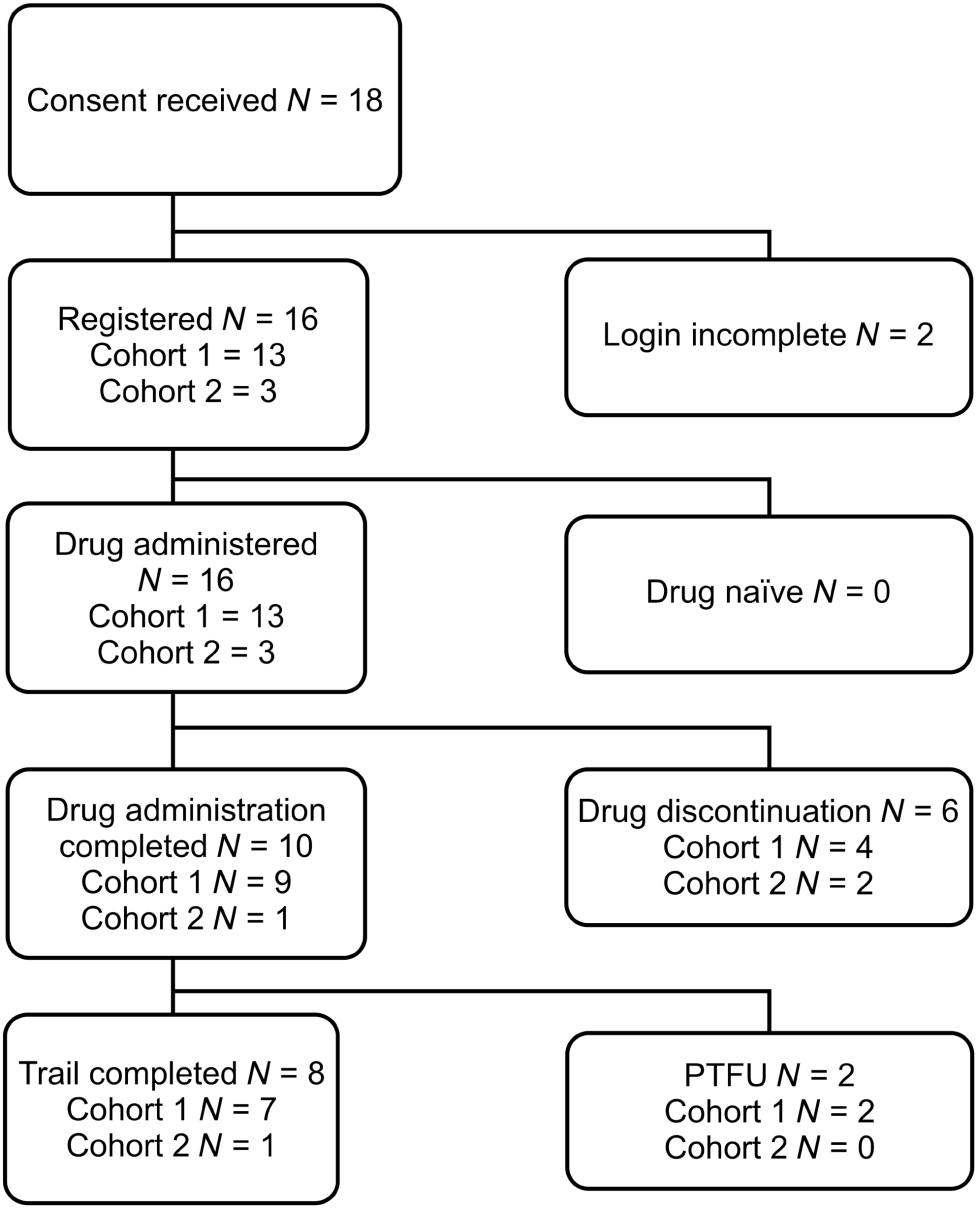
Patient disposition. PTFU, post‐treatment follow‐up.

In cohort 1, all enrolled patients (*n* = 13) received BV. Nine patients completed up to 16 cycles of BV administration, while four discontinued treatment. In cohort 2, one patient completed the treatment and two discontinued. A total of nine patients in both cohorts completed the study without discontinuation during PTFU (Figure 2).

### 3.2 Patient characteristics

The baseline characteristics and demographics of patients included in FAS1 and FAS2 are presented in Table 1. The median age (range) was 54.0 (32–76) and 52.5 (32–78) years for FAS1 and FAS2, respectively. The total mSWAT scores were 43.05 ± 40.344 and 36.18 ± 39.026 for FAS1 and FAS2, respectively. Most patients received systemic chemotherapy as their primary treatment (85% in FAS1 and 75% in FAS2), followed by radiation therapy and phototherapy (Table 1).

**TABLE 1:** Patient demographics and clinical characteristics at baseline.

			FAS1*N* = 13	FAS2*N* = 16
Gender	Male		8 (62%)	10 (63%)
	Female		5 (38%)	6 (38%)
Age (years), mean (SD)			52.8 (13.15)	54.1 (13.40)
≤64			11 (85%)	13 (81%)
≥65			2 (15%)	3 (19%)
Total mSWAT score		Mean (SD)	43.05 (40.34)	36.18 (39.02)
Total BSA of lesions (erythema, mm^2^)		Mean (SD)	18.76 (26.79)	15.42 (25.02)
Total BSA of lesions (phase, mm^2^)		Mean (SD)	3.96 (6.26)	3.38 (5.74)
Total BSA of lesions (mass, mm^2^)		Mean (SD)	4.09 (4.72)	3.50 (4.43)
Primary disease name	CD30 + MF		8 (62%)	8 (50%)
	pcALCL		5 (38%)	5 (31%)
	Lymphomatoid papulosis		0	2 (13%)
	PCGDTCL		0	1 (6%)
Primary disease TNMB classification (MF cases)	T			
		T3	7 (88%)	7 (88%)
		T4	1 (13%)	1 (13%)
	N	N0	3 (38%)	3 (38%)
		N1	1 (13%)	1 (13%)
		N3	1 (13%)	1 (13%)
		Nx	3 (38%)	3 (38%)
		M0	8 (100%)	8 (100%)
	B	B0	8 (100%)	8 (100%)
	Staging	IIB	6 (75%)	6 (75%)
		IIIA	1 (13%)	1 (13%)
		IVA2	1 (13%)	1 (13%)
Primary disease TNM classification (pcALCL, other cases)	T			
		T2a	0	1 (13%)
		T3a	1 (20%)	1 (13%)
		T3b	4 (80%)	6 (75%)
	N	N0	3 (60%)	5 (63%)
		N2	2 (40%)	3 (38%)
		M0	5 (100%)	8 (100%)
History of treatment for primary disease		SDT: Steroids	5 (38%)	6 (38%)
		SDT: Chemotherapy	1 (8%)	1 (6%)
		SDT: Radiation therapy	7 (54%)	7 (44%)
		SDT: Phototherapy	7 (54%)	8 (50%)
		SDT: Other	2 (15%)	3 (9%)
		Systemic therapy: Bexarotene	8 (62%)	9 (56%)
		Systemic therapy: Chemotherapy (methotrexate)	4 (31%)	4 (25%)

	Systemic therapy: Chemotherapy (other)	11 (85%)	12 (75%)

	Systemic therapy: Retinoids	3 (23%)	4 (25%)

	Systemic therapy: Immunotherapy	3 (3%)	3 (19%)

	Systemic therapy: Histone deacetylase inhibitor	1 (8%)	1 (6%)

	Procedure	1 (8%)	2 (13%)
Medical history			8 (62%)	10 (63%)
		Breast cancer	1 (8%)	1 (6%)
		Cataract	1 (8%)	1 (6%)
		Cerebral infarction	1 (8%)	1 (6%)
		Constipation	1 (8%)	1 (6%)
		Asteatotic eczema	1 (8%)	1 (6%)
		Inguinal hernia	0	1 (6%)
		Pituitary tumor	1 (8%)	1 (6%)
		Excessive fat in blood	1 (8%)	1 (6%)
		Steroid diabetes	1 (8%)	1 (6%)
12‐lead ECG	Abnormal findings		2 (15%)	2 (13%)
ECOG PS	0		9 (69%)	12 (75%)
	1		2 (15%)	2 (13%)
	2		2 (15%)	2 (13%)
Histopathological examination (central determination) CD30	positive		9 (69%)	11 (69%)

Abbreviations: BSA, body surface area; ECG, electrocardiogram; ECOG, Eastern Cooperative Oncology Group; FAS, full analysis set; MF, mycosis fungoides; mSWAT, modified severity‐weighted assessment tool; pcALCL, primary cutaneous anaplastic large‐cell lymphoma; PCGDTCL, primary cutaneous gamma‐delta T‐cell lymphomas; PS, performance status; SD, standard deviation; SDT, skin directed therapy; TNM, tumor node metastasis; TNMB, tumor‐node‐metastasis‐blood.

### 3.3 Efficacy

#### 3.3.1 Primary endpoint: ORR4 (IRF determination)

Using an exact binomial test (one‐tailed) for the null hypothesis of 12%, the point estimate of ORR4 by IRF in the main analysis of the primary endpoint was 69.2% (95% CI 38.6–90.9) for FAS1 and 62.5% (95% CI 35.4–84.8) for FAS2, which was statistically significant (*P* < 0.0001) (Table 2).

**TABLE 2:** Summary of efficacy in FAS population.

Population analyzed	*n*/*N*	ORR4 point estimate (95% CI)	Exact binomial test^a^
			*P* value
FAS1	9/13	69.2% (38.6–90.9)	<0.0001
FAS2	10/16	62.5% (35.4–84.8)	<0.0001

Abbreviations: CI, confidence interval; FAS, full analysis set; ORR4, objective response rate lasting at least 4 months.

^a^ Null hypothesis is 12%, one‐tailed test.

Similarly, ORR4 (IRF determination) in the subanalysis of mFAS1 and mFAS2 was 55.6% (5/9, 95% CI 25.1–83.1) and 45.5% (5/11, 95% CI 20.0–72.9, *P* = 0.0021 and *P* = 0.0061), respectively.

In an exploratory analysis, the IRF‐based point estimate of ORR4 showed no notable differences in the stratified assessment of sex, age, and total mSWAT score for FAS1 and FAS2. However, ORR4 was 100% in the primary disease categories of pcALCL and ECOG PS 2 (Table 3).

**TABLE 3:** Exploratory analysis of ORR4 (IRF determination).

Population analyzed		FAS1			FAS2		
		*n*/*N*	Point estimate	95% CI	*n*/*N*	Point estimate	95% CI
Gender	Male	6/8	75.0%	34.9–96.8	6/10	60.0%	26.2–87.8
	Female	3/5	60.0%	14.7–94.7	4/6	66.7%	22.3–95.7
Age (year)	≤64	8/11	72.7%	39.0–94.0	9/13	69.2%	38.6–90.9
	≥65	1/2	50.0%	1.3–98.7	1/3	33.3%	0.8–90.6
Primary illness	MF	4/8	50.0%	15.7–84.3	4/8	50.0%	15.7–84.3
	pcALCL	5/5	100.0%	47.8–100.0	5/5	100.0%	47.8–100.0
	Other	0	0.0%	—	1/3	3.3%	0.8–90.6
ECOG PS	0–1	7/11	63.6%	30.8–89.1	8/14	57.1%	28.9–82.3
	2	2/2	100.0%	15.8–100.0	2/2	100.0%	15.8–100.0
Total mSWAT score at screening^a^	Below median	4/5	80.0%	28.4–99.5	5/8	62.5%	24.5–91.5
	Above median	5/8	62.5%	24.5–91.5	5/8	62.5%	24.5–91.5
Staging (MF cases)	IIB	3/6	50.0%	11.8–88.2	3/6	50.0%	11.8–88.2
	IIIA	0/1	0.0%	0.0–97.5	0/1	0.0%	0.0–97.5
	IVA2	1/1	100.0%	2.5–100.0	1/1	100.0%	2.5–100.0

^a^ Total mSWAT median score was calculated using both FAS1 and FAS2 at screening.

Abbreviations: 95% CI, 95% confidence interval; ECOG, Eastern Cooperative Oncology Group; FAS, full analysis set; MF, mycosis fungoides; mSWAT, modified severity‐weighted assessment tool; pcALCL, primary cutaneous anaplastic large‐cell lymphoma; PS, performance status.

### 3.4 Secondary endpoints

#### 3.4.1 Response rates

According to the Kaplan–Meier method, the cumulative response rates at 16 months were 69.2% and 72.7% for FAS1 and FAS2, respectively.

The point estimate response rates in FAS1 and FAS2 were 53.8% (7/13, 95% CI 25.1–80.8, *P* = 0.0003) and 56.3% (9/16, 95% CI 29.9–80.2, *P* < 0.0001), respectively, as determined by the investigators using GRS.

The cutaneous response rates in FAS1 and FAS2 were 61.5% (8/13, 95% CI 31.6–86.1) and 62.5% (10/16, 95% CI 35.4–84.8), respectively, as determined by the physician using mSWAT.

The IRF evaluated cutaneous response rates using mSWAT for other efficacy endpoints, with comparable rates of 69.2% (9/13, 95% CI 38.6–90.9) and 62.5% (10/16, 95% CI 35.4–84.8) in FAS1 and FAS2, respectively.

Both the physician's opinion at the implementing institution and the IRF concluded that the point estimate of ORR1 for GRS and mSWAT assessments was 69.2%. Notably, there were no reports of PD or recurrence. The overall response rate based on GRS was 31% for CR, 38% for PR, and 31% for SD. The response rate for CR + PR reached 69% and 100% for CR + PR + SD, respectively. The total mSWAT percentage change from baseline (mean ± SD) was 8.83 ± 17.56 in cycle 1, −21.20 ± 29.06 in cycle 2, and remained below baseline throughout PTFU. The lymph node CR rate was 80%, the CR + PR rate was 80%, and the CR + PR + SD rate was 100% for FAS2 and was 67%, 67%, and 83% for FAS1, respectively.

### 3.5 Survival

Owing to the small number of events (1/13 in FAS1 and 1/16 in FAS2) and the high number of censored cases (12/13 in FAS1 and 15/16 in FAS2), the median OS could not be determined. At 11 months, the cumulative survival rate in FAS1 and FAS2 was 100% (95% CI, 100.0–100.0) using the Kaplan–Meier method. At 33 months, the cumulative survival rates were 90.0% (47.3–98.5) in FAS1 and 90.9% (50.8–98.7) in FAS2.

**FIGURE 3:**
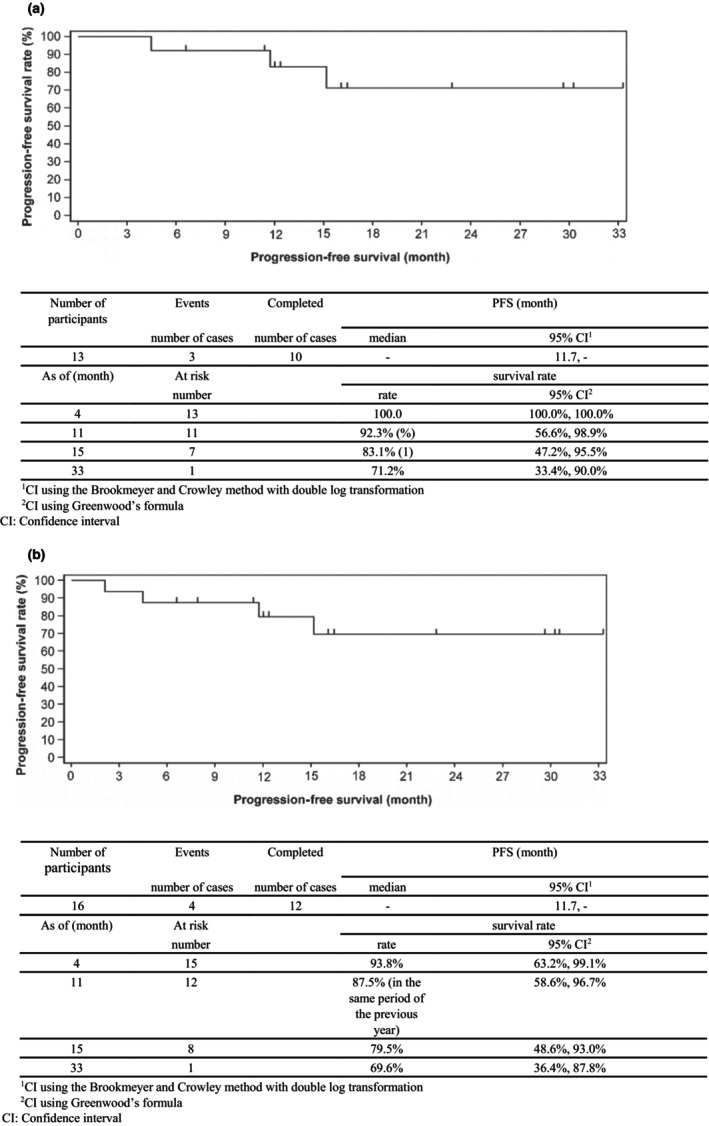
Progression‐free survival (IRF determination, GRS). (a) FAS1. (b) FAS2. FAS, full analysis set; GRS, global response score; IRF, independent review forum.

The median PFS could not be determined based on GRS (IRF determination; number of events: 3/13, number of censored cases: 10/13 in FAS1; number of events: 4/16, number of censored cases: 12/16). At 15 months, the cumulative survival rates assessed using the Kaplan–Meier method were 83.5% in FAS1 and 79.5% in FAS2. At 33 months, the PFS rates were 71.2% and 69.6% in FAS1 and FAS2, respectively (Figure 3).

### 3.6 Duration of response

**FIGURE 4:**
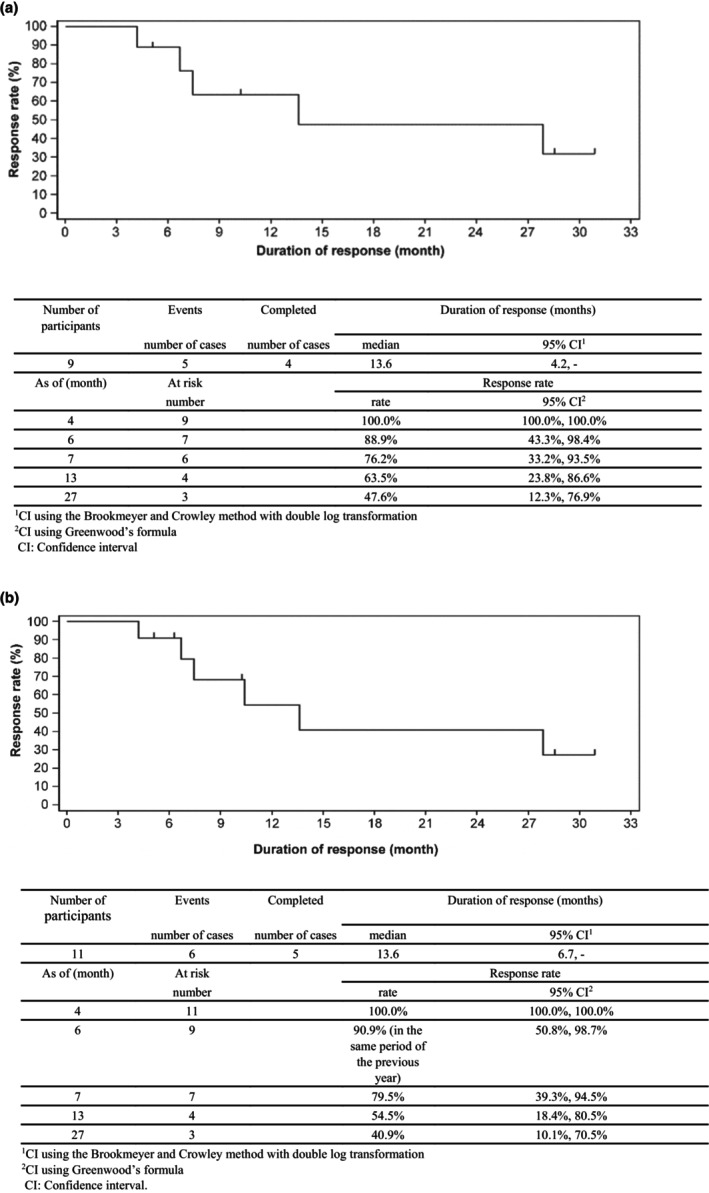
Duration of response for skin lesions as determined by a physician at the performing institution, mSWAT. (a) FAS1 (response cases in mSWAT). (b) FAS (response cases in mSWAT). FAS, full analysis set; mSWAT, modified version of the Medical Severity Assessment Tool.

In FAS1, median duration of response (DOR) for skin lesions was 13.6 months (6.7 months to inestimable). Five of nine cases experienced an event (loss of response), and four discontinued. Similarly, in FAS2, median DOR was 13.6 months (6.7 months to inestimable). Six of 11 cases experienced an event, and five were censored. The response rate showed a comparable pattern: 100.0% (100.0–100.0) at 4 months, 54.5% (23.8–77.4) at 13 months, and 40.9% (14.0–66.6) at 27 months (Figure 4).

### 3.7 Time to response

The median time to response for skin lesions (as determined by the site's physicians) using mSWAT was 1.8 months in FAS1 and FAS2. The median time to response among responders (nine patients in FAS1 and 11 patients in FAS2) was 1.74 months.

### 3.8 Remission of the lesion and quality‐of‐life assessment

The percentage change in body surface area (BSA) indicated no significant difference from cycles 1 to 6 for erythema. Cycle 7 significantly decreased (mean ± SD = −7.9 ± 94.5). Other parameters did not change significantly from cycles 1 to 4, but cycle 5 observed a decreasing trend (mean ± SD = −57.1 ± 59.6), remaining below baseline except for one parameter, which increased by 34.8 ± 13.7 in cycle 10. Mass was 37.7 ± 91.7 after cycle 1 and then decreased to −14.0 ± 110.4 after cycle 2. PTFU was low, except for 12 weeks at 77.9 ± 289.7 and the last visit at 40.69 ± 299.36. There were no blood lesions in patients with MF.

Baseline DLQI scores in FAS1 decreased after cycle 2 (−1.3 ± 3.4) and EOT/ET (−0.8 ± 2.22) with a consistent decrease in 12‐week PTFU. Change in DLQI scores from baseline in FAS1 was −5.6 ± 6.6 in patients who reached ORR4 and −1.8 ± 2.87 in SD patients.

There was no significant correlation between CD30 expression rate and ORR4 attainment (Supporting Information Figure S1 and Table S3). %BSA for erythema, facies, mass, and lesion, mSWAT score, and T‐cell receptor β chain reconstitution did not correlate with ORR4 or ORR1 (Supporting Information Figure S2).

### 3.9 Safety

Adverse events in SAF1 and SAF2 were identified as adverse reactions. One death occurred outside the collection of adverse events due to PD. Significant adverse events affected 100% and 94% of patients in SAF1 and SAF2, respectively, with 15% and 19% of patients discontinuing treatment due to adverse reactions (Supporting Information Table S4).

In SAF1 and SAF2, 15% or more of patients experienced grade 3 adverse reactions, with peripheral neuropathy occurring in 23% and 19% of patients, respectively. Eosinophilia and fever were observed in 15% or more of patients in SAF1, whereas all other grade 3 reactions in SAF1 and SAF2 occurred in less than 15% of patients (one or two cases) (Table 4). Grade 4 adverse reactions were neutropenia (8% and 6%, respectively) in SAF1 and SAF2. There were no grade 5 adverse reactions. Severe adverse reactions, such as eosinophilia, inguinal hernia, fever, neutropenia, hyperuricemia, peripheral neuropathy, acute kidney injury, and pulmonary toxicity, occurred in 10% of patients (one case each).

**TABLE 4:** Aggregated ≥grade 3 adverse events.

SOC	SAF1		SAF2	
	Number of cases (%)	Number of occurrences	Number of patients (%)	Number of occurrences
PT				
Number of participants	13		16	
Whole	8 (62%)	19	9 (56%)	20
Nervous system disorders	3 (23%)	3	3 (19%)	3
Peripheral neuropathy	3 (23%)	3	3 (19%)	3
Blood and lymphatic system disorders	2 (15%)	2	2 (13%)	2
Hypereosinophilia	2 (15%)	2	2 (13%)	2
General and systemic disorders and conditions at the site of administration	2 (15%)	3	2 (13%)	3
Generation of heat	2 (15%)	3	2 (13%)	3
Gastrointestinal disorder	1 (8%)	1	2 (13%)	2
Inguinal hernia	1 (8%)	1	2 (13%)	2
Hepatobiliary system disorders	1 (8%)	1	1 (6%)	1
Abnormal liver function	1 (8%)	1	1 (6%)	1
Infectious and parasitic diseases	1 (8%)	1	1 (6%)	1
Osteomyelitis	1 (8%)	1	1 (6%)	1
Clinical examination	1 (8%)	5	1 (6%)	5
Neutropenia	1 (8%)	3	1 (6%)	3
Decreased white blood cell count	1 (8%)	2	1 (6%)	2
Metabolic and nutritional disorders	1 (8%)	1	1 (6%)	1
Hypertriglyceridemia (hypertriglyceridemia, hypertriglyceridemia)	1 (8%)	1	1 (6%)	1
Renal and urinary tract disorders	1 (8%)	1	1 (6%)	1
Acute renal failure	1 (8%)	1	1 (6%)	1
Respiratory, thoracic, and mediastinal disorders	1 (8%)	1	1 (6%)	1
Lung toxicity	1 (8%)	1	1 (6%)	1

Abbreviations: PT, preferred term; SAF, safety analysis set; SOC, system organ class.

There were five adverse reactions in three patients who discontinued BV treatment. Pulmonary toxicity was a serious adverse reaction that mildly resolved after discontinuation of BV. Two other adverse reactions, peripheral neuropathy and liver function abnormality, were of non‐serious grade 3 severity. The remaining two events were of grade 2 severity.

## 4 DISCUSSION

This phase I/II study evaluated the efficacy and safety of BV in Japanese patients with relapsed or refractory CD30+ CTCL, divided into two cohorts: cohort 1 (participants with CD30+ MF or pcALCL) and cohort 2 (participants with CD30+ LPDs other than those in cohort 1). The efficacy and safety of BV for treating refractory MF/SS and CD30+ LPDs are consistent with previous phase II or III studies.^18^, ^19^, ^20^, ^21^ To the best of our knowledge, this is the first multicenter phase I/II clinical trial to assess BV for the treatment of CD30+ CLs in Japan.

In this study, the primary and secondary endpoint efficacy analyses yielded promising results. Based on IRF determination, the ORR4 for the primary endpoints in FAS1 and FAS2 was 69.2% and 62.5%, respectively, significantly exceeding expected values (12%, *P* < 0.0001). Conventional treatments for CTCL often have response rates of 30%–50%. Although chemotherapy results in a higher response rate, DOR is often short. Other experimental drugs showed an ORR of 31% in Japanese patients with CTCL.^22^ Our findings were consistent with ORR4 (57.3% in the BV arm) reported in the Alcanza trial, which also used IRF assessment as the primary endpoint,^20^ phase 2 studies of BV in CTCL (70%–73%),^18^, ^19^ and a recent Spanish registry, which reported an ORR of 67% in patients with advanced‐stage MF and CD30 LPDs.^23^ Furthermore, most patients in this study (60%) maintained PR or CR for at least 4 months after initiating BV treatment following the initial response. In addition, consistent findings across secondary endpoints in this study support the primary findings.

In the Alcanza trial, the 3‐year OS with BV was estimated to be 64.4% (median follow‐up was 45.9 months) and the median PFS was 16.7 months.^21^ The median PFS of a novel monoclonal antibody in an ongoing study was 7.7 months.^24^ In the present study, patients had a 100% survival rate at 11 months and a 90% survival rate at 3 months. Similarly, the Kaplan–Meier PFS estimates evaluated using GRS and mSWAT revealed a sustained PFS rate of 100% at 2–4 months for FAS1 and FAS2. These findings suggest that treating patients with relapsed or refractory CD30+ MF, pcALCL, and other CD30+ LPDs with BV as a subsequent chemotherapy regimen may increase long‐term response rates and extend PFS beyond 4 months.

The median time to skin lesion response, as assessed by a physician, was 1.8 months in our study, indicating a rapid response onset. In addition, the median length of skin lesion response indicated by the physician was 13.6 months. In patients with relapsed or refractory CD30+ MF, pcALCL, and other CD30+ LPDs, BV improved skin lesions with notable improvements occurring within approximately 2 months of study enrollment and lasting until the fourth month. Furthermore, there was a trend toward a 30% improvement rate at 30 months, indicating that a subset of patients showed continued improvement.

The efficacy ratings for lymph node involvement (80% for CR + PR) in our study suggest that BV is effective in treating such lesions, highlighting its potential as a treatment option for a subset of patients with CD30+ LPDs. However, the number of patients with lymph node involvement was limited in this study. A high remission rate and persistent improvement in skin lesions over time, as evidenced by mSWAT and %BSA scores, suggest a gradual improvement in skin condition. Analysis of change from baseline in DLQI scores based on the time of CR determination showed significant improvement in DLQI scores, notably in patients with ORR4 and SD, suggesting the most pronounced overall impact. ORR4 stratified analysis by IRF determination revealed no significant differences. Although differences were observed in primary disease, ECOG performance status, and staging, the sample size did not provide adequate statistical power for IRF determination.

Studies examining the response to BV in different types of NHL did not find a significant association between CD30 expression and response to BV treatment.^25^, ^26^ In contrast, research in CTCL has suggested that higher CD30 expression (≥5%) is linked to a greater likelihood of response compared to lower expression (<5%). However, our findings align with those of the Alcanza trial^20^ and a European retrospective study,^27^ suggesting the efficacy of BV treatment irrespective of CD30 expression levels.

The safety profile of BV observed in this study is consistent with previous studies.^18^, ^19^, ^20^, ^21^, ^22^ The overall incidence of adverse events decreased over time, particularly in cycles 1 and 2, which had the highest incidence. These findings suggest a higher incidence of adverse events during the initial cycles, underscoring the importance of implementing appropriate strategies for managing early‐stage TEAE, such as considering treatment discontinuation.

Our study has a few limitations. The small sample size of this study may have affected the generalizability of the results. The follow‐up period in the study may not have been sufficient to capture long‐term effects or potential late‐onset adverse events associated with BV treatment.

## 5 CONCLUSION

In conclusion, this phase I/II study found that BV had a promising effectiveness and safety profile in Japanese patients with relapsed or refractory CD30+ CTCL. Studies with a larger sample size and longer follow‐ups are needed to validate our findings and provide insights into the therapeutic benefits of BV.

## FUNDING INFORMATION

Takeda Pharmaceutical Company Limited (IISR Grant X2504) funded the design and conduct of the study; data collection, management, analysis, and interpretation of the data; manuscript preparation, review, and approval; editorial services and the decision to submit the manuscript for publication through an investigator‐initiated clinical trial. This trial was funded by Takeda Pharmaceutical Company Limited. The experimental drug used in this clinical trial was also provided by Takeda Pharmaceutical Company Limited.

## CONFLICT OF INTEREST STATEMENT

Yoji Hirai received research grants, consultancy fees, speaker fees, and funds for staff members from Takeda Pharmaceutical Company Limited. Tomomitsu Miyagaki, Riichiro Abe, and Shin Morizane are members of Editorial Board of the *Journal of Dermatology* and a coauthor of this article. To minimize bias, they were excluded from all editorial decision‐making related to the acceptance of this article for publication.

## Supporting information


Supporting Information Table S1.
Supporting Information Table S2.
Supporting Information Table S3.
Supporting Information Table S4.
Supporting Information Figure S1.
Supporting Information Figure S2.
