# Supplementary material for: Phase I/II clinical trial of brentuximab vedotin for pretreated Japanese patients with CD30‐positive cutaneous T‐cell lymphoma
Source: J Dermatol. 2024 Jun 14;51(8):1037–49. doi: 10.1111/1346-8138.17324 (PMC11483954; doi:10.1111/1346-8138.17324)
Supplement: Supplementary file 1 — Supporting Information Table S1. Supporting Information Table S2. Supporting Information Table S3. Supporting Information Table S4. Supporting Information Figure S1. Supporting Information Figure S2. [file JDE-51--s001.docx]

**Phase I/II Clinical Trial of Brentuximab Vedotin for Pretreated Japanese Patients with CD30-positive Cutaneous T-cell Lymphoma**

Running title: Brentuximab Vedotin in Japanese CTCL patients

Yoji Hirai^1^, Jun Sakurai^2^, Shiho Yoshida^2^, Takashi Kikuchi^2^, Toshiharu Mitsuhashi^2^, Tomoko Miyake^1^, Taku Fujimura^3^, Riichiro Abe^4^, Hiroki Fujikawa^4^, Hikari Boki^5^, Hiraku Suga^5^, Sayaka Shibata^5^, Tomomitsu Miyagaki^5^, Takatoshi Shimauchi^6^, Eiji Kiyohara^7^, Yoshio Kawakami^1^, Shin Morizane^1^

^1^Department of Dermatology, Okayama University Graduate School of Medicine, Dentistry, and Pharmaceutical Sciences, Okayama, Japan

^2^Center for Innovative Clinical Medicine, Okayama University Hospital, Okayama, Japan

^3^Department of Dermatology, Tohoku University Graduate School of Medicine, Sendai, Japan

^4^Department of Dermatology, Niigata University, Niigata, Japan

^5^Department of Dermatology, Tokyo University, Tokyo, Japan

^6^Department of Dermatology, Hamamatsu University School of Medicine, Hamamatsu, Japan

^7^Department of Dermatology, Osaka University, Osaka, Japan

Corresponding author:

Dr. Yoji Hirai,

Department of Dermatology, Graduate School of Medicine, Dentistry, and Pharmaceutical Sciences, Okayama University,

2-5-1 Shikata-cho, Kita-ku, Kita-ku, Okayama 700-8558, Japan

Tel: +81-86-235-7282

Fax: +81-86-235-7283

Email: [gmd20033@s.okayama-u.ac.jp](mailto:gmd20033@s.okayama-u.ac.jp)

**Supplementary Appendix**

Table of contents

Table S1. Principal investigators and clinical trial management organization

Table S2. Selection of the study population

Table S3. Summary of adverse events in the SAF population

Table S4. ORR attainment and CD30 expression

Figure S1. Correlation with CD30 expression and ORR

Figure S2. Correlation with %BSA and ORR

**Table S1.** Principal investigators and clinical trial management organization

|  | Function |
| --- | --- |
| Yoji Hirai, Department of Dermatology, Okayama University Hospital  2-5-1 Shikata-cho, Kita-ku, Okayama City, Okayama 700-8558, Japan  TEL: 086-235-7282 | Coordination among investigators in a multicenter clinical trial and coordination on interpretation of questions in the study protocol that arise during the clinical trial. The coordinating investigator shall submit the clinical trial notification on behalf of the investigator |
| Yoji Hirai, Department of Dermatology, Okayama University Hospital  2-5-1 Shikata-cho, Kita-ku, Okayama City, Okayama 700-8558, Japan  TEL: 086-235-7282 | Adhere to the study protocol and take responsibility for all medical decisions related to the clinical trial. Ensure that adequate medical care is provided to the subject for all adverse events related to the clinical trial during the subject's participation in the trial and thereafter. Provide sufficient information, guidance and supervision to the investigators and collaborators regarding the clinical trial. |
| Eiji Kiyohara, Department of Dermatology, Osaka University Hospital  2-15 Yamadaoka, Suita-shi, Osaka 565-0871, Japan  TEL: 06-6879-5111 |  |
| Hikari Bohki, Department of Dermatology, The University of Tokyo Hospital  7-3-1 Hongo Bunkyo-ku Tokyo 113-8656, Japan  TEL: 03-3815-5411 |  |
| Taku Fujimura, Department of Dermatology, Tohoku University Graduate School of Medicine  1-1 Seiryo-cho, Aoba-ku, Sendai 980-8574, Japan  TEL: 022-717-7000 |  |
| Takatoshi Shimauchi, Department of Dermatology, Hamamatsu University School of Medicine  1-20-1 Handayama, Higashi-ku, Hamamatsu city, Shizuoka, 431-3192, Japan  TEL 053-435-2111 |  |
| Riichiro Abe, Department of Dermatology, Niigata University Medical and Dental Hospital  754 Asahimachidori Ichibancho, Chuo-ku, Niigata 951-8520, Japan  TEL: 025-223-6161 (main) |  |
| Jun Sakurai  Center for New Medical Research and Development, Okayama University Hospital  2-5-1 Shikata-cho, Kita-ku, Okayama City, Okayama 700-8558, Japan  TEL: 086-235-6510　FAX: 086-235-6505 | Confirm that the work related to this clinical trial is being conducted in accordance with the GCP ordinance and the clinical trial protocol. |
| Yoshihiro Sato  Center for New Medical Research and Development, Okayama University Hospital  2-5-1 Shikata-cho, Kita-ku, Okayama City, Okayama 700-8558, Japan  TEL: 086-235-7515　FAX: 086-235-6505 | Evaluate from the viewpoint of quality assurance whether this clinical trial is conducted in compliance with the GCP ordinance, the study protocol, and the operating procedures. |
| Jun Irie  Center for New Medical Research and Development, Okayama University Hospital  2-5-1 Shikata-cho, Kita-ku, Okayama City, Okayama 700-8558, Japan  TEL: 086-235-6037 | The Data Management Plan, the electronic case report form (eCRF), the electronic clinical laboratory information capture (EDC) system design, data checking and fixing, and data validation/validation will be performed. |
| Toshiharu Mitsuhashi  Center for New Medical Research and Development, Okayama University Hospital  2-5-1 Shikata-cho, Kita-ku, Okayama City, Okayama 700-8558, Japan  TEL: 086-235-6037 | Ensure that statistical principles are properly applied to the study. Prepare an analysis plan, conduct statistical analysis, and summarize the results in a report. |

**Table S2.** Selection of the study population

| **Inclusion Criteria**  The investigators determined the eligibility of subjects based on the following criteria Patients who met all the requirements of the following items were included in this study.  1. Patients who have obtained free and voluntary consent based on the explanatory and consent documents approved by the IRB, etc.  Consent should be obtained before any testing related to the clinical trial is performed.  2. Male or female Japanese patients 20 years of age or older at the time of consent  3. Patients with CD30-positive lymphoproliferative disorders (including pcALCL and CD30+MF) confirmed histologically by institutional pathology  Cohort 1: Patients with histologically diagnosed pcALCL, CD30+MF  Cohort 2: Patients diagnosed with CD30-positive lymphoproliferative disorders other than cohort 1  4. Patients who had received at least one cycle of systemic therapy (IFN, bexarotene, vorinostat, anti-tumor chemotherapy, etc.) with inadequate response in the previous treatment. In pcALCL, patients who had received radiation therapy previously and had an inadequate response were also included. Insufficient response is usually judged as worsening of the disease after one or more cycles of systemic therapy, and is determined by the physician based on a comprehensive evaluation of clinical symptoms, imaging findings, and other factors.  5. Patients with ECOG PS less than or equal to 2  6. Patients who have recovered from all severities of clinically significant adverse effects of prior systemic therapy to NCI-CTCAE v5.0 Grade 1 or less  7. Patients who meet the following criteria at the time of screening test  (1) ANC > 1,500/μL  (2) Platelet count > 75,000/µL  (3) Total bilirubin < 1.5 x ULN  (4) AST, ALT < 3×ULN  AST and ALT are eligible up to 5 times ULN or less if the elevation is known to be due to liver involvement.  (5) Creatinine clearance or calculated creatinine clearance > 40 mL/min  (6) Hb > 8 g/dL  8. Patients who agree to use appropriate contraceptive methods from the time of screening until EOT/ET  Premenopausal female patients of childbearing potential who have agreed to use a medically approved method of contraception from the time of signing the informed consent document until 6 months after the last dose of study drug or, in the case of male patients of childbearing potential, during the period of study drug administration and until 6 months after the last dose of study drug  9. Patients undergoing anticancer therapy who can be washed out for 3 weeks after obtaining consent and before administration of the study drug  10. Patients receiving antibody therapy, immunoglobulin-based immunotherapy, or other monoclonal antibody therapy who are able to washout 12 weeks after obtaining consent and prior to administration of study drug. However, the washout period may be set to 3 weeks if the investigator determines that the subject needs to start receiving the investigational drug as soon as possible due to a rapid worsening of the disease. |
| --- |
| **Exclusion criteria**  The investigators excluded from the study those who met any of the following criteria  1. sALCL Merger  2. Complications of SS or B2 disease  Peripheral blood sesary cell counts shall be determined by institutional determination. If the abnormal lymphocyte count exceeds 750/uL, the subject will be measured according to the central judgment procedure, and the study coordinating physician will make a comprehensive judgment of the results to determine the eligibility of the subject.  3. Patients with any of the following cardiovascular conditions or laboratory values within 6 months prior to enrollment  (1) myocardial infarction  (2) NYHA Class III or IV heart failure  (3) ECG findings of cardiac arrhythmia, CHF, angina pectoris, or acute ischemia or uncontrolled cardiovascular findings including clinically significant conduction system abnormalities  4. History of other primary malignancies that have not been in remission for at least 3 years  However, there is no 3-year limit for cases of completely resected intraepithelial cancers such as non-melanoma skin cancer with intraepithelial lesions, intraepithelial cervical cancer on biopsy, and intraepithelial tumors of the cervix on cytological examination.  5. Known active brain/meningeal disease including signs or symptoms of PML  6. Patients with previously confirmed HIV infection. For subjects who have never been tested for HIV, HIV testing is not required to determine eligibility for this study.  7. Known hepatitis B surface antigen positive or known or suspected active hepatitis C infection  8. Severe active, systemic viral, bacterial, or fungal infection requiring systemic therapy (e.g., intravenous antibacterial, antiviral, or antifungal agents) within 1 week prior to study drug administration (oral antibiotics for prophylaxis are permitted).  Patients with uncomplicated staphylococcal infections (any species of staphylococci) are eligible if they have been controlled with a certain dose of antimicrobial therapy (but not intravenous antimicrobial agents) prior to screening.  9. Patients who have received antibody-directed or immunoglobulin-based immunotherapy (e.g., immunoglobulin replacement, other monoclonal antibody therapy) within 12 weeks prior to study drug administration  10. Patients who received systemic or local corticosteroid therapy for the treatment of CTCL within 3 weeks prior to the administration of the investigational drug. However, local topical therapy less than or equal to Strong's class that is ongoing prior to enrollment and is SD during the screening period may be used in combination.  11. Patients who received systemic administration of 15,000 IU or more of vitamin A or etretinate per day for the treatment of CTCL within 3 weeks prior to study drug administration  12. Patients with pre-existing or concomitant hypersensitivity to recombinant proteins, mouse proteins, or drug components contained in the formulation  13. Pregnant or lactating female patients, or female patients with a positive serum pregnancy test during the screening period or urine pregnancy test on day 1 of any cycle  14. Patients who have received radiation therapy, other SDT, or other investigational therapy within 3 weeks prior to study drug administration  SDT includes the following; topical steroid and ACNU therapy, ultraviolet light therapy (NB-UVB, excimer light, PUVA), local irradiation, etc.  15. Pancreatitis or significant risk factors (e.g., history of pancreatitis, uncontrolled hyperlipidemia, excessive alcohol consumption, uncontrolled diabetes, biliary disease and elevated triglyceride levels, or concomitant use of drugs associated with pancreatic toxicity) or amylase level > ULN at screening and Lipase level > 3 x ULN or higher  16. Patients previously treated with brentuximab vedotin  17. Other patients whom the investigator deems inappropriate as subjects |

**Table S3.** Summary of adverse events in the Safety analysis set

|  | SAF1 Number of cases (%) | SAF2 Number of cases (%) |
| --- | --- | --- |
| Number of participants | 13 | 16 |
| Adverse events | 13 (100) | 16 (100) |
| Side effects | 13 (100) | 16 (100) |
| Serious adverse events | 5 (38) | 5 (31) |
| Serious side effects | 5 (38) | 5 (31) |
| Significant adverse events^#^ | 13 (100) | 15 (94) |
| Adverse events leading to discontinuation of treatment | 2 (15) | 3 (19) |
| Side effects that led to discontinuation of treatment | 2 (15) | 3 (19) |

^#^significant adverse events were defined as peripheral neuropathy, hematologic toxicity, infection, injection-related reactions, PML, Stevens-Johnson syndrome, toxic epidermal necrolysis, tumor disruption syndrome, acute pancreatitis, pulmonary toxicity, liver toxicity, hyperglycemia, gastrointestinal disturbances

| **Table S4.** ORR attainment and CD30 expression | | | | | | | | | | | | | |
| --- | --- | --- | --- | --- | --- | --- | --- | --- | --- | --- | --- | --- | --- |
| **Case  no.** | **Disease type** | **ORR4 attainment** | **ORR1 attainment** | **No. of  CD30 cells 1** | **No. of  CD30 cells 2** | **No. of  CD30 cells 3** | **Total lymphocytes 1** | **Total lymphocytes 2** | **Total lymphocytes 3** | **CD30 expression rate 1** | **CD30 expression rate 2** | **CD30 expression rate 3** | **Mean CD30  expression rate** |
|  |  |  |  |  |  |  |  |  |  |  |  |  |  |
| 1 | CD30+ MF | Yes | Yes | 0 | 0 | 1 | 2011 | 2162 | 1565 | 0.0% | 0.0% | 0.1% | **0.0%** |
| 2 | CD30+ MF | Yes | Yes | 275 | 255 | 201 | 2157 | 2080 | 2847 | 12.7% | 12.3% | 7.1% | **10.7%** |
| 3 | CD30+ MF | No | No | 108 | 139 | 101 | 1695 | 2038 | 1638 | 6.4% | 6.8% | 6.2% | **6.5%** |
| 4 | CD30+ MF | No | No | 834 | 13 | 201 | 2872 | 1449 | 2456 | 29.0% | 0.9% | 8.2% | **12.7%** |
| 5 | PCGDTCL | No | No | 218 | 184 | 392 | 3013 | 2981 | 2839 | 7.2% | 6.2% | 13.8% | **9.1%** |
| 6 | CD30+ MF | No | No | 115 | 170 | 157 | 3267 | 2999 | 3011 | 3.5% | 5.7% | 5.2% | **4.8%** |
| 7 | LyP | Yes | Yes | 39 | 14 | 7 | 1801 | 2098 | 1147 | 2.2% | 0.7% | 0.6% | **1.1%** |
| 8 | pcALCL | Yes | Yes | 5 | 2 | 4 | 2267 | 2864 | 2669 | 0.2% | 0.1% | 0.1% | **0.1%** |
| 9 | pcALCL | Yes | Yes | 2871 | 1891 | 2769 | 3479 | 3187 | 3721 | 82.5% | 59.3% | 74.4% | **72.1%** |
| 10 | pcALCL | Yes | Yes | 1410 | 838 | 904 | 2988 | 2517 | 2419 | 47.2% | 33.3% | 37.4% | **39.3%** |
| 11 | CD30+ MF | Yes | Yes | 7 | 8 | 16 | 2580 | 2295 | 2857 | 0.3% | 0.3% | 0.6% | **0.4%** |
| 12 | CD30+ MF | Yes | Yes | 216 | 226 | 139 | 1761 | 2995 | 1325 | 12.3% | 7.5% | 10.5% | **10.1%** |
| 13 | CD30+ MF | No | No | 132 | 94 | 139 | 230 | 122 | 160 | 57.4% | 77.0% | 86.9% | **73.8%** |
| 14 | pcALCL | Yes | Yes | 2 | 0 | 6 | 2187 | 1073 | 2760 | 0.1% | 0.0% | 0.2% | **0.1%** |
| 15 | pcALCL | Yes | Yes | 2206 | 2024 | 2157 | 3172 | 3044 | 3008 | 69.5% | 66.5% | 71.7% | **69.2%** |
| 16 | LyP | No | No | 606 | 241 | 292 | 3791 | 3592 | 3178 | 16.0% | 6.7% | 9.2% | **10.6%** |

MF, mycosis fungoides; ORR1, objective global response lasting at least 1 month; ORR4, objective global response lasting at least 4 months; pcALCL, primary cutaneous anaplastic large-cell lymphoma; PCGDTCL, primary cutaneous gamma-delta T-cell lymphomas; LyP, lymphomatoid papulosis; SAF, safety analysis population.

**Figure S1.** Correlation with CD30 expression and ORR

**
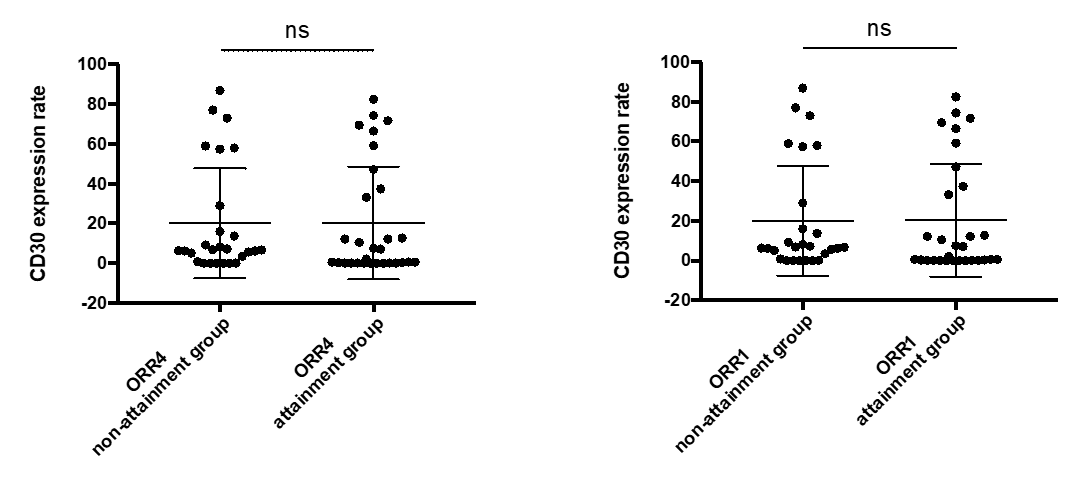
**

For each case, CD30 immunostaining was performed on skin biopsy tissue from the lesion at screening, and images were obtained from three random locations (20x objective field of view), and the CD30 expression rate (number of CD30 positive cells/total lymphocytes) was examined.

ns, non significant; ORR1, objective global response lasting at least 1 month; ORR4, objective global response lasting at least 4 months.

**Figure S2.** Correlation with %BSA and ORR

**
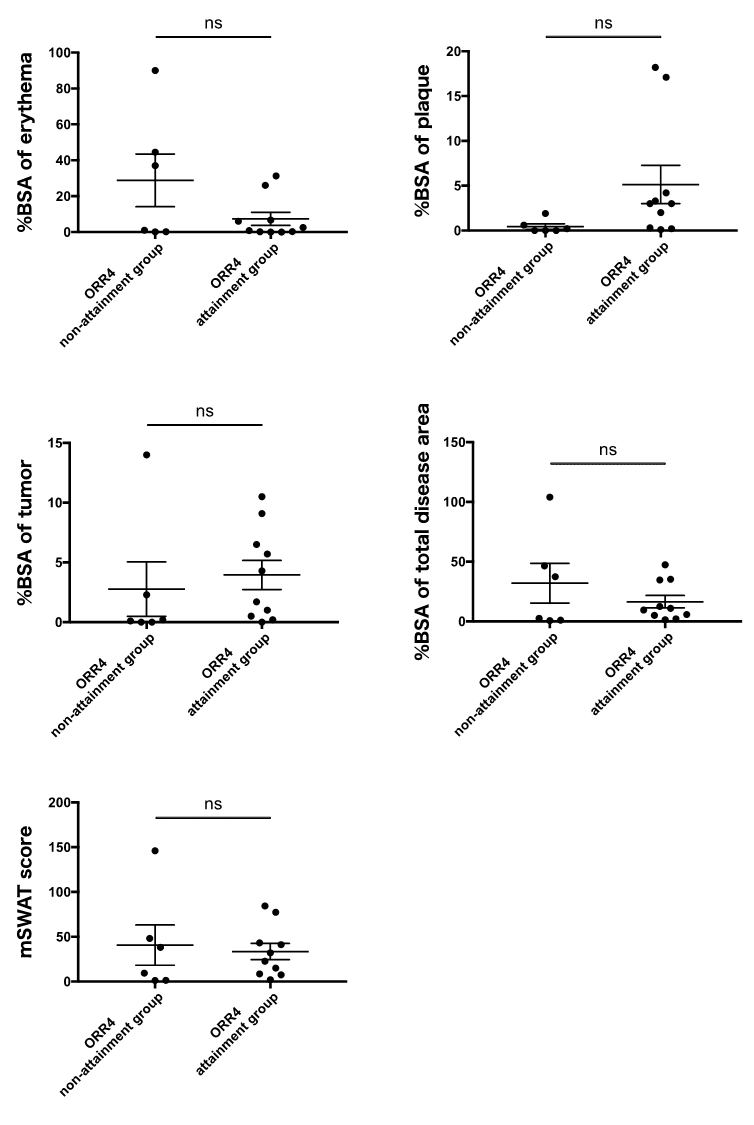
**

BSA, body surface area; mSWAT, modified severity-weighted assessment tool; ns, non significant; ORR4, objective global response lasting at least 4 months.
